# Changing the policy for intermittent preventive treatment with sulfadoxine–pyrimethamine during pregnancy in Malawi

**DOI:** 10.1186/s12936-017-1736-9

**Published:** 2017-02-20

**Authors:** Chikondi A. Mwendera, Christiaan de Jager, Herbert Longwe, Kamija Phiri, Charles Hongoro, Clifford M. Mutero

**Affiliations:** 1 School of Health Systems and Public Health, Institute for Sustainable Malaria Control (UP ISMC), University of Pretoria, Private Bag X363, Pretoria, 0001 South Africa; 2 Mailman School of Public Health, ICAP at Columbia University, Pretoria, South Africa; 3 School of Public Health and Family Medicine, College of Medicine, University of Malawi, Blantyre, Malawi; 4 Population Health, Health Systems and Innovation, Human Sciences Research Council (HSRC), Pretoria, South Africa; 5 International Centre of Insect Physiology and Ecology (ICIPE), P.O. Box 30772, Nairobi, Kenya

**Keywords:** Malaria, Pregnancy, Sulfadoxine–pyrimethamine, Policy change, Malawi

## Abstract

**Background:**

The growing resistance of *Plasmodium falciparum* to sulfadoxine–pyrimethamine (SP) treatment for uncomplicated malaria led to a recommendation by the World Health Organization for the use of artemisinin-based combination therapy. Inevitably, concerns were also raised surrounding the use of SP for intermittent prevention treatment of malaria during pregnancy (IPTp) amidst the lack of alternative drugs. Malawi was the first country to adopt intermittent prevention treatment with SP in 1993, and updated in 2013. This case study examines the policy updating process and the contribution of research and key stakeholders to this process. The findings support the development of a malaria research-to-policy framework in Malawi.

**Methods:**

Documents and evidence published from 1993 to 2012 were systematically reviewed in addition to key informant interviews.

**Results:**

The online search identified 170 potential publications, of which eight from Malawi met the inclusion criteria. Two published studies from Malawi were instrumental in the WHO policy recommendation which in turn led to the updating of national policies. The updated policy indicates that more than two SP doses, as informed by research, overcome the challenges of the first policy of two SP doses only because of ineffectiveness by *P. falciparu*m resistance and the global lack of replacement drugs to SP for IPTp.

**Conclusion:**

International WHO recommendations facilitated a smooth policy change driven by motivated local leadership with technical and financial support from development partners. Policy development and implementation should include key stakeholders and use local malaria research in a research-to-policy framework.

## Background

The resistance of the malaria parasite to anti-malarial drugs has led to expensive policy changes in many countries causing strain on available resources [1]. Although the World Health Organization (WHO) is instrumental in guiding health policy development, contextual factors unique to different countries need to be assessed before adopting and implementing these recommendations [2].

Policy development is a tedious process that requires an understanding of the institutional and individual actors and of the context in which the process occurs [3]. Walt and Gilson [4] described this relationship when they developed a health policy framework that explores the context, content, and processes in which actors are engaged. Andersen [5] developed a framework for understanding the policy process incorporating problem identification, agenda setting, policy formulation, policy adoption, policy implementation and policy evaluation.

Malawi established its National Malaria Control Programme (NMCP) in 1984 [6]. The NMCP based their first five-year implementation plan (1985–1989) on WHO recommendations for malaria control in areas with proven chloroquine (CQ) resistance in Africa [7]. One of its policies was to provide CQ chemoprophylaxis to special groups of individuals including pregnant women. In response to the growing evidence of CQ resistance, studies assessed alternative drugs to replace CQ. Malawi invested tremendously in research on malaria in pregnancy and became the first country to adopt intermittent preventive treatment in pregnancy with sulfadoxine–pyrimethamine (IPTp-SP) in 1993 [8, 9]. The policy recommended that pregnant women should receive two doses during pregnancy with the first dose being given at the first antenatal visit after the first trimester of pregnancy (typically 16th to 22nd week of gestation) and the second dose at the beginning of the third trimester (between 28 and 34 weeks of gestation) [10]. Over time, growing *P. falciparum* resistance to SP for the treatment of uncomplicated malaria led to the WHO recommendation of switching to artemisinin-based combination therapy (ACT) [11] and related concerns were raised about the efficacy of SP for IPTp. The WHO convened an Evidence Review Group (ERG) on IPTp-SP in 2012 which reviewed various research evidence relevant to updating the IPTp policy. The ERG acknowledged that more than two doses of IPTp-SP would be more beneficial than the usual two doses that were previously administered [8]. The ERG recommended that IPTp-SP be given at each antenatal visit, with the first dose given early in the second trimester and subsequent doses given at monthly intervals up to the time of delivery. Following these recommendations, in 2013 the NMCP in Malawi adapted its IPTp-SP policy by recommending that women should receive at least three doses of SP during pregnancy [12].

The process of updating the IPTp-SP policy was examined with the aim of understanding policy development. The role of stakeholders and relevant research evidence during the policy development in Malawi was also assessed.

### Conceptual framework

This case study forms part of a larger effort to understand policy development and the role of relevant research in this process in order to develop a framework that can facilitate the use of evidence from malaria research for policy formulation in Malawi. One important aspect of policy analysis is to understand the involvement of stakeholders and research in the process while considering the various factors that govern the need for the policy [13]. This study was conceptualized on the premise that different factors besides overwhelming evidence may influence policy development. The Walt and Gilson policy analysis framework [4] stipulates that aside from content analysis, the actors, processes and the context in which policy change occurs are required for policy analysis. The policy analysis was supplemented by the Andersen’s model of policy cycle [5].

## Methods

Mixed methods in form of a systematic review of published evidence, a review of key documents and key informant in-depth interviews (KIIs) were utilized in the policy analysis to enable triangulation.

### Systematic review

This method aimed to establish the availability of local evidence likely to be used in the policy change process. Relevant articles were sought by searching the references of all reviewed articles. Combinations of the following specific key words relating to IPTp-SP were searched by using the medical subject heading (MESH) strategy: sulfadoxine–pyrimethamine (SP), Fanasil, pyrimethamine drug combination, pregnancy, and Malawi. Articles published between 1993 and 2012 were searched to capture all studies conducted in Malawi related to IPTp-SP from inception to the time of the policy update. These studies were assumed to provide timely evidence and were more likely to be included in the policy development process. The following combinations were used during the search: (“pregnancy” [MeSH Terms] OR “pregnancy” [All Fields]) AND (“fanasil, pyrimethamine drug combination” [Supplementary Concept] OR “fanasil, pyrimethamine drug combination” [All Fields] OR “sulfadoxine–pyrimethamine” [All Fields]) AND (“Malawi” [MeSH Terms] OR “Malawi” [All Fields]) AND (“1993/01/01” [PDAT]: “2012/12/31” [PDAT]). We searched the MEDLINE (Ovid), PubMed, Scopus and Cochrane Library databases.

### Selection criteria

From the articles identified by the systematic review above, studies selected for analysis were based on the following criteria: (1) conducted in Malawi between 1993 and 2012; (2) evaluating two doses of IPTp-SP; (3) evaluating three or more IPTp-SP doses; (4) assessing two versus three or more IPTp-SP doses. The selection was limited to studies assessing the optimal response of *P. falciparum* infection to IPTp-SP by excluding studies conducted on HIV-positive women. HIV infection reduces the ability of a pregnant woman to control the malaria infection resulting in a suboptimal response to IPTp-SP [14]. Two independent co-authors, CM and HL, judged the eligibility of the studies and resolved disagreements by consensus.

### Document review

Available documents such as reports, circulars, directive letters and minutes from meetings conducted during the policy development process were sought to provide a forum for triangulation, to verify the stakeholders and to verify important dates and events throughout the process. WHO IPTp policy documents [8, 15] and local IPTp-SP policy documents [12] were reviewed to examine the extent to which they referenced research evidence during policy development.

### Key informant interviews

KIIs with key stakeholders involved in policy development comprising malaria researchers/advisors, policy makers, and programme/project coordinators were conducted. Interviewees participated in the policy updating process, and their views were considered to capture what transpired and general experiences on the change. Purposive sampling identified key informants. Fifteen individuals were identified and interviewed. Six interviewees were senior malaria-in-pregnancy researchers and advisors, three interviewees were policy makers and six interviewees were programme/project coordinators. Table 1 summarizes the experience, expertise and role played by each key informant (KI) during the policy changes.

**Table 1:** Details of key informants (KIs) involved in the policy update for intermittent preventative treatment during pregnancy with sulfadoxine-pyrimethamine (IPTp-SP) for malaria in Malawi

KI	Sex	Expertise	Experience	Role
1	Male	Malaria epidemiologist	Over 15 years in malaria research	Researcher/advisor
2	Male	Medical epidemiologist	Over 10 years in malaria research	Researcher/advisor
3	Female	Malaria epidemiologist	Over 30 years in malaria research	Researcher/advisor
4	Male	Clinician and malaria epidemiologist	Over 40 years in malaria research	Researcher/advisor
5	Male	Malaria epidemiologist	Over 10 years malaria research	Researcher/advisor
6	Male	Senior malaria scientist	Over 40 years in malaria research	Researcher/advisor
7	Female	Clinical epidemiologist	6 years	Policy maker
8	Male	Malaria in pregnancy coordinator	Over 5 years in malaria research	Policy maker
9	Male	Chief of health services	Five years	Policy maker
10	Female	Malaria in pregnancy coordinator	Over 5 years in malaria research	Programme/project coordinator
11	Male	Malaria advisor	15 years	Programme/project coordinator
12	Male	Malaria advisor	5 years	Programme/project coordinator
13	Male	Policy development and analysist	4 years	Programme/project coordinator
14	Male	Malaria advisor	5 years	Programme/project coordinator
15	Male	Malaria program specialist	20 years	Programme/project coordinator

The principal investigator conducted all the interviews, probing and exploring in-depth issues based on the conceptual framework of the study. The interviews were conducted in English using a semi-structured interview tool.

### Themes covered in the in-depth interviews

The participants were asked to narrate their memories of the policy process by contemplating the question: *“Can you please describe the process by which the IPTp*-*SP policy change occurred in Malawi?”*

Interviews covered specific themes that included: (1) context in which the policy occurred; (2) opportunities during the policy process; (3) challenges encountered during the policy process and (4) lessons learned.

### Data management and analysis

The recordings were transcribed and coded based on the themes, and the software Nvivo 11 was used to organize the data, while verbatim quotes illustrated concepts, supported conclusions and brought reality to the situation. The Giorgi’s phenomenological approach, which focuses on the experiences of participants with shared life experiences, was used. This approach documents the findings from the interviewee’s point of view, collecting their descriptions of their lived world on the interpretation of the meaning of the described phenomena [16].

## Results

### Systematic review

**Fig. 1:**
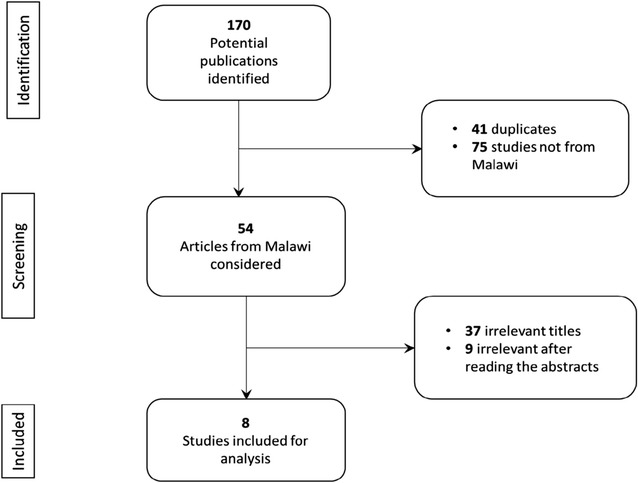
A flow chart of the selection process for studies reviewed

One hundred and seventy potential publications were identified using database searches of which eight publications from Malawi were selected using the inclusion criteria and subsequently reviewed (Fig. 1).

### Description of the publications

Eight studies that met the inclusion criteria were reviewed (Table 2). Verhoeff et al. [17] measured the parasite prevalence in mothers who received one, two, or three SP doses during pregnancy and the associated incidence of low birth weight (LBW) in infants. Although there was no significant difference in peripheral or placental blood parasite prevalence, the mean birthweights of infants were higher, resulting in a decrease of LBW babies born to mothers that received two or more SP doses. Taylor et al. [18] explored the link between IPTp-SP, the presence of resistant parasite at delivery and multiple measures of adverse delivery outcome. Receiving SP as IPTp did not raise pregnancy-associated malaria despite increasing prevalence and fixation of SP-resistant *P. falciparum.* Although LBW prevalence was lower (11.8%) in the full IPTp group than in the suboptimal group (P = 0.48), the difference was not significant. Taylor et al. [18] recommended the modified regimen of IPTp-SP for comprehensive antenatal care. Rogerson et al. [19] assessed the relationship between the number of IPTp-SP doses and various health indicators. Placental malaria prevalence decreased from 31.9%, in women who did not receive SP, to 22.8% in women with ≥2 SP-doses, while LBW prevalence decreased from 23% (no IPT) to 10.3% (IPTp-SP) in the two groups of women. Rogerson et al. [19] recommended that IPTp-SP should be continued based on the positive impact, but that researchers continuously evaluate treatment. Filler et al. [14] assessed the efficacy of monthly SP compared to the two doses of SP in preventing placental malaria in both HIV positive and negative women. HIV negative women who received a monthly dose of SP had a lower (2.3%) incidence of placental malaria compared to women who received two doses (6.3%). Filler et al. [14] recommended that areas of intense falciparum transmission should adopt a monthly IPTp-SP regimen. This study was included in the meta-analysis that led to the WHO policy recommendation [15, 20].

**Table 2:** Characteristics of intermittent preventative treatment during pregnancy with sulfadoxine–pyrimethamine (IPTp-SP) related studies conducted in Malawi

No.	Publication	Study objective and type	Study population	Study type	Approach	Results found	Conclusion
1	Verhoeff et al. [17] March 1993 to June 1994 study period	Compared and evaluated parasite prevalence, anaemia and LBW in mothers who received one, two or three doses of SP during pregnancy, and the incidences of LBW in the infants	575 pregnant women attending antenatal facility at Chikwawa district hospital in Malawi	Interventional, longitudinal study	Assessment was in women who received one, two or three doses of SP during pregnant	No significant difference in parasite prevalence in peripheral or placental blood between women who received one or two SP doses although multigravidea with two dose SP had higher haemoglobin concentrations than those who received one dose (*P* = 0.009). The mean birthweights were higher, and incidence of LBW lower in babies born to primi- and multi-gravidea who had received two or three doses of SP than those from women who received just one dose (*P* < 0.03 for each)	SP use was not associated with maternal side-effects or perinatal complications and that multiple doses of SP during pregnancy will lead to a highly significant reduction in the incidence of LBW.
2	Taylor et al. [18] July 1997 to August 2006 study period	Explored relationships between IPTp-SP, the presence of resistant parasite at delivery, and multiple measures of adverse delivery outcome, including parasite densities, placental histology, maternal haemoglobin concentration and birth weight	177 genotyping and antenatal data of pregnant women delivering at Queens Elizabeth Central Hospital in Blantyre, Malawi	A serial cross section analysis	SP receipt records were obtained from antenatal clinical cards, peripheral and placental blood obtained, and a subset of 25% of available sample from women with positive peripheral blood thick smear were tested for genotyping	Women who received full IPTp with SP (≥2 doses) had lower peripheral (*P* = 0.018) and placental (*P* < 0.0001) parasite densities than women who received suboptimal IPTp (<2 doses), mean birthweight in the full IPTp group of 2892 g compared to 2776 g in the suboptimal group (*P* = 0.086), or LBW prevalence of 11.8% in the full IPTp group compared to 15.8% in the suboptimal group (*P* = 0.481)	The receipt of SP as IPTp did not raise PAM morbidity despite the increasing prevalence and fixation of SP-resistant *P. falciparum* haplotypes and therefore SP may be used in modified IPTp regimens as a component of comprehensive antenatal care
3	Rogerson et al. [19] July 1997 to April 1999 study period	Assessed operational effectiveness of SP by examining the relationship between number of doses of SP prescribed in antenatal clinic and health indicators	1044 women attending the maternity unit at Queen Elizabeth Central Hospital in Blantyre, Malawi	Clinical study	Samples from peripheral and placental blood were collected and tested. With 251 women having received no SP, 555 received 2SP-dose, and 238 received ≥2 SP-doses	SP was associated with a decrease in placental malaria prevalence from 31.9% with no SP to 22.8% with ≥2 SP-doses. Decreased prevalence of LBW from 23% in women not receiving SP to 10.3% in the group receiving ≥2 SP-doses, while maternal and cord blood malaria prevalence and mean cord blood haemoglobin concentrations did not differ with SP usage	IPTp-SP had a positive impact on some indicators while improved implementation and surveillance are critical
4	Filler et al. [14] October 2002 to March 2005 study period	Determined the efficacy of monthly SP compared to the 2-dose regimen in preventing placental malaria in both HIV positive and negative women. (Results of HIV negative women only are considered in this review)	432 HIV negative women were randomized (216 received 2-dose SP while 216 received monthly SP)	Randomized, non-blinded study	Participants were randomized into either receiving 2-dose SP or monthly SP	In the HIV negative group 2.3% who received monthly SP compared to 6.3% who received 2-dose SP had placental malaria (RR, 0.37)	Monthly IPTp-SP is more efficacious than a 2-dose regimen in preventing placental malaria and that monthly IPTp-SP should be adopted in areas of intense transmission of falciparum malaria
5	Luntamo et al. [21] December 2003 to October 2006 study period	Examined the potential to prevent preterm deliveries and LBW through intensified gestational intermittent preventive treatment containing antibiotics against malaria and reproductive tract infections	1320 women with uncomplicated second trimester pregnancies at Lungwena Health center, Mangochi, Malawi	A single-center, randomized, partially placebo controlled, outcome assessor-blinded clinical trial	The compared interventions included a standard 2-dose SP as a control group (436), monthly SP (441), and monthly SP combined with two doses of azithromycin (AZI-SP) (443)	Preterm incidence was 17.9% in the controls, 15.4% in the monthly SP group (*P* = 0.32), and 11.8% in the AZI-SP group (*P* = 0.01). While comparing with the controls the AZI-SP group had a risk ratio of 0.61 (*P* = 0.02) and the monthly SP group had a risk ratio of 0.71 (*P* = 0.09) for LBW	The incidence of preterm delivery and LBW can in some conditions be reduced by treating pregnant women with monthly SP and two dose azithromycin
6	Luntamo et al. [22] December 2003 to October 2007 study period	Assessed the effect of monthly SP and AZI-SP treatments on peripheral malaria parasitemia at delivery in a population of both HIV-positive and –negative women of all gravidities using the PCR-methodology	484 samples from women with uncomplicated second trimester pregnancies at Lungwena Health center, Mangochi, Malawi	A single-center, randomized, partially placebo controlled, outcome assessor-blinded clinical trial	The compared interventions included a standard 2-dose SP as a control group (162), monthly SP (151), and monthly SP combined with two doses of azithromycin (AZI-SP) (171)	Comparing with controls, the monthly group had a risk ratio of 0.33 (P < 0.001) and in the AZI-SP group 0.23 (P < 0.001) for malaria at delivery. While in only HIV-negative women the corresponding figures were 0.26 (P < 0.001) in the monthly SP group ad 0.24 9 (P < 0.001) in the AZI-SP group for malaria at delivery	Increasing the frequency of SP doses during pregnancy improves efficacy against malaria at delivery among HIV-negative women, including a population of both HIV-negative and –positive women of all gravidities
7	Luntamo et al. [23] December 2003 to October 2006 period of study	Assessed the ability to reduce foetal and neonatal growth faltering through IPTp of malaria and reproductive tract infections with monthly SP, alone or with two doses of azithromycin	1320 women with uncomplicated second trimester pregnancies at Lungwena Health center, Mangochi, Malawi	A randomized, partially placebo controlled, outcome assessor-blinded clinical trial	Participants received either two doses of SP (control) (436), SP monthly (441), or SP monthly and azithromycin (1 g) twice (AZI-SP) (443)	Babies in the AZI-SP group were on average 140 g heavier at birth and 0.6 cm longer at four weeks of age than in the control group	Monthly IPTp-SP regimen provided to all pregnant women is likely to increase mean birthweight and length at four weeks of age in malaria holoendemic areas and adding azithromycin to the regimen seems to increase the benefit in reduction of fetal and neonatal growth faltering
						Babies in the monthly SP group were on average 80 g heavier and 0.3 cm longer than in the control group	
						Compared to controls, the AZI-SP group had a relative risk of 0.61 LBW, 0.60 stunting, and 0.48 underweight at four weeks of age	
						Compared to controls, the monthly SP group had a relative risk of 0.71 LBW, 1.02 stunting, and 0.87 underweight	
8	Gutman et al. [24] March and August 2010 study period	Assessed the effectiveness of IPTp-SP	703 HIV-negative women were enrolled at Machinga district hospital in Malawi	Cross-sectional delivery survey	Assessment was made in 22% (154) of women who received <2 SP-doses and those that received ≥2 SP-doses	IPTp-SP was associated with a dose-dependent protective effect on composite birth outcomes in primigravidae of an adjusted prevalence ratio of 0.50, 0.30, and 0.18 for 1, 2, and ≥3 doses respectively when compared to 0 doses	IPTp-SP did not reduce the frequency of placental infection but was associated with improved birth outcomes and that IPTp-SP should still continue to be administered although alternative strategies and drugs should be explored

Several similar studies have assessed the efficacy of the monthly SP dose. Luntamo et al. [21] compared the effect of monthly SP, or monthly SP and two doses of azithromycin (AZI-SP) to the standard 2-SP regimen in preventing preterm deliveries and LBW. Preterm incidence was 17.9% in controls (2-SP), 15.4% in the monthly group (P = 0.32) and 11.8% in the AZI-SP group (P = 0.01). There was a lower risk of LBW in the AZI-SP group (0.61, P = 0.02) and the monthly SP group (0.71, P = 0.71) compared to the control group. Luntamo et al. [21] concluded that AZI-SP reduces the incidence of preterm delivery and LBW under certain conditions. Luntamo et al. [22] compared the effect of monthly SP, or monthly SP and two doses of azithromycin (AZI-SP) and a standard 2-SP dose (control) on malaria at delivery. HIV-negative women that received a monthly dose of SP (0.26, P < 0.0001) and those that received the AZI-SP regimen (0.249, P < 0.0001) had a significantly lower risk of malaria compared to the control group. Luntamo et al. [22] recommended that frequency of SP doses during pregnancy should be increased; these recommendations were included in the meta-analysis leading to amended WHO policy recommendations [15, 20]. Luntamo et al. [23] compared the effect of monthly SP, or AZI-SP and a standard 2-SP dose (control) on foetal and neonatal growth. Pregnant women who received monthly SP had babies with heavier mean birthweights that were taller at four weeks of age, and the addition of azithromycin further increased the benefits in reducing growth faltering. Gutman et al. [24]. assessed the effectiveness of IPTp-SP on placental infection and composite birth outcomes. Their findings showed that IPTp-SP was associated with a dose-dependent protective effect on composite birth outcomes but did not reduce the frequency of placental infection. They recommended that IPTp-SP be given while exploring alternative strategies and drugs.

### Document review

Policy documents were examined to assess how research evidence was used and included in WHO documents [8, 15] and the local treatment policy [12]. The WHO documents used a variety of evidence from across the globe upon which they based their policy formulation. Evidence from Malawi was instrumental in agenda setting [9, 25] and policy development [14, 18, 19, 21–23, 26]. The local treatment policy document did not have a formal reference section which hampered the assessment of used evidence. However, the authors of the policy document acknowledged the importance of the WHO recommendations during its development.

### Stakeholders and their roles during the IPTp-SP policy change

The Ministry of Health (MOH) (represented by the NMCP and the Reproductive Health Directorate (RHD)), the National Malaria Advisory Committee (NMAC); Malaria Care, Clinton Health Access Initiative (CHAI), WHO; Support for Service Delivery Integration-Services (SSDI-services), malaria researchers, and PMI/USAID were primary stakeholders in the policy change process. Stakeholders gave technical advice, developed guidelines, reviewed and edited guidelines, trained health workers, implemented policies in health facilities, and provided financial support for conferences and other resources (Table 3).

**Table 3:** Summary of activities provided by key stakeholders involved in the policy updating process for intermittent preventative treatment during pregnancy with sulfadoxine-pyrimethamine (IPTp-SP) for malaria in Malawi

Stakeholder	Main responsibility	Role in policy change
NMCP	Development of malaria policies, and implementation of malaria programs	Drafting of guidelines, leading the process, and finalization of guidelines
RHD	Implementation of reproductive health services in the MOH	Drafting of the guidelines, and policy implementation
SSDI-services	Effective integration and delivery of quality services under the Malawi Essential Health Package (EHP), and to strengthen the national health system in line with the National Health Sector Strategic Plan for 2011–2016	Coordination of activities, drafting of guidelines, finalizing, printing, dissemination of guidelines, and training of health workers
WHO	Provision of technical advice and recommendation	Overseeing of the whole process in accordance to WHO recommendations
PMI/USAID	Provision of technical and financial support for the NMCP	Provided financial support for all activities and provided technical advice
NMAC	Provide expert opinion to the NMCP in policy and programme development	Vetting and final approval of the guidelines
Malaria Care	Provision of malaria diagnostic and treatment services	Training of health workers
CHAI	Strengthening of integrated health systems	Revision of case management guidelines, training of health workers
Malaria researchers	Conducting malaria research to provide evidence and guide policy formulation	Provided technical review of evidence and guidelines

*NMCP* National Malaria Control Programme, *RHD* Reproductive Health Directorate, *SSDI* support for service delivery integration, *PMI* President’s Malaria Initiative, *USAID* United States Agency for International Development, *NMAC* National Malaria Advisory Committee, *CHAI* Clinton Health Access Initiative, *MOH* Ministry of Health, *WHO* World Health Organization

### The process of change

The policy change began in July 2012 when the WHO ERG made a recommendation to the Malaria Policy Advisory Committee (MPAC) for an interim policy on IPTp-SP [8]. The recommendation was adopted after an assessment of a systematic review and meta-analysis [20]. The ERG based their recommendations on findings that associated three or more SP doses for IPTp with increased mean birth weight and reduced risk of LBW births. Based on the reviewed evidence, the ERG recommended IPTp-SP for all pregnant women with the first dose administered at antenatal visits as early as possible in the second trimester, and the subsequent doses spaced no less than four weeks apart up to the time of delivery [8]. Following this meeting in October 2012, WHO updated its IPTp-SP policy, and in April 2013 a policy brief was issued to support dissemination and urge national health authorities to adopt and implement the new recommendations.

### Steps for IPTp-SP policy change in Malawi

Based on emerging literature and WHO recommendations, the NMCP updated malaria treatment guidelines that incorporated rectal and injectable artesunate, malaria rapid diagnostic tests (RDTs) for quick diagnosis of uncomplicated malaria, and the new IPTp-SP policy. Malawian policy makers took the opportunity to adapt the IPTp-SP policy given the challenges experienced during the implementation of the previous policy. The implementation of the previous policy was hampered by health workers’ confusion surrounding the delivery of the second dose of SP [27]. The updated WHO recommendations do not specify the number of doses, but highlight that SP should be provided to pregnant women at each scheduled antenatal visit after the first trimester up until the time of delivery. The adapted Malawi IPTp-SP policy indicates that pregnant women should receive at least three doses of SP after the first trimester and with the last dose given close to the time of delivery. The new recommendation was strategically planned to coincide with the WHO initiative of integrating IPTp-SP into focused antenatal care (FANC) services that recommend at least four scheduled antenatal care visits [15].

In May 2013, the NMAC convened to vet the treatment guidelines before the Minister of Health and the Secretary for Health approved the new guidelines. Initially, Malaria in Pregnancy (MIP) formed part of the treatment guidelines in Malawi, and it was deemed vital to isolate and develop specific guidelines for MIP. In June 2013, a group comprising of the NMCP, RHD, and PMI/USAID, coordinated by the SSDI-services, convened for one week to develop MIP guidelines and revise the MIP training manual for health workers following the WHO recommendations. The guidelines were approved in July 2013 [12].

**Fig. 2:**
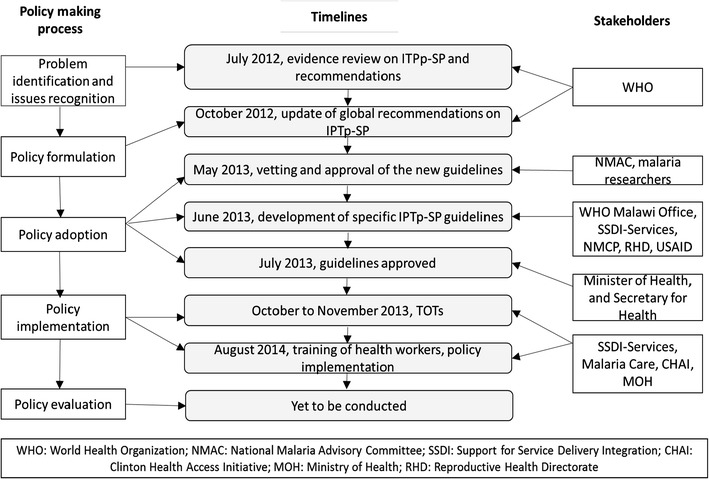
The policy making process, timeline of events and stakeholders involved in the policy updating process for intermittent preventative treatment during pregnancy with sulfadoxine-pyrimethamine (IPTp-SP) for malaria in Malawi

From October to November 2013, two Trainer of Trainers (TOTs) workshops were conducted in the northern and central eastern zones of the country to orient TOTs on the updated malaria case management guidelines and new IPTp-SP policy. The TOTs were immediately required to roll out training sessions for other health workers in their work places. Despite this requirement, training sessions for health workers only commenced in August 2014. The delay in transition was due to a change in the per diem policy by the government through the Office of President and Cabinet (OPC) on all Developing Partner (DP) programmes in Malawi. The per diem policy changed regarding direct payments to service providers for costs such as accommodation. Participants did not receive sitting allowances and were not paid if they conducted duties for which they received a salary during the training session. These adjustments meant that participants were not able to pay for necessities if they travelled to different training venues. Logistical issues were resolved by conducting training sessions in areas where most of the participants were situated, and the new policy was implemented in August 2014. Evaluation of the new IPTp-SP policy is yet to be conducted. Figure 2 illustrates the timeline of policy change process, and the roles played by stakeholders based on Andersen’s model of policy change [5].

### Context of policy change

In 2007, Malawi changed its treatment policy for uncomplicated malaria from SP to artemether–lumefantrine. The change of drug regimen was complicated as it attracted many activities which required approval by the Ministry of Health. The new treatment guidelines for IPTp-SP were introduced in 2013 and was uncomplicated by comparison. Updating the policy did not draw any official launch since it was approved along with the treatment policy. This offered an enabling environment for a smooth transition of the IPTp-SP policy change as narrated below:

*“In fact there is no need for launching when it’s a revised policy but if it’s a new policy that’s when the launching comes in” (Programme/Project Coordinator)*

The policy update aimed to overcome a flaw of the old 2-IPTp-SP policy. Health workers were confused about the exact time to give the second dose mainly due to late antenatal attendance by women leading to the programme failing to meet the 80% Roll Back Malaria coverage target [27]. The new policy was adapted to address this challenge as highlighted below:

*“We noted a big challenge that there was a misunderstanding in terms of application by the service providers because it [the policy] was saying at 13* *weeks give the first dose of SP then the second dose at 28* *weeks…so people just complied to those dates…so if one comes at 18th week or 20th week then [they] will not be given SP and who comes at 32* *weeks would not be given the second dose…so the previous guidelines made some limitation and that was our main challenge for low coverage but this policy change to 3 doses or more means that a pregnant woman can get IPTp even after 36* *weeks…there are no restrictions so that’s one of the advantages”. (Programme/Project Coordinator)*

The global lack of an alternative drug to replace SP for IPTp meant that increasing the doses, as stipulated by evidence, was the only tangible alternative. The challenges of coverage (as highlighted above), lack of an alternative drug and new evidence on dosage increment provided an ideal environment for IPTp-SP policy change.

*“We do not have any other alternative that’s why they are recommended that we should just use 3 or more doses but studies by College of Medicine are underway to explore other drugs”. (Programme/Project Coordinator)*

### Opportunities for smooth policy transition

Participants revealed that the availability of technical and funding support from partners (USAID/PMI and SSDI-services) supported the completion of policy updating process. This was acknowledged as follows:

*“Funding component and technical support was assured by the project…but also PMI who are the funders of the project…a malaria section…I think they have been very supportive….I think they wanted this to succeed as such their pressure made it easier for us to move forward”. (Programme/Project Coordinator)*

The relatively low cost of SP facilitated smooth policy transition. The low cost of SP meant that increasing doses would not incur a heavy cost, and that partners were willing to fund the policy implementation.

*“SP is frankly pretty [an] inexpensive drug…so we can usually cover the entire need for a few hundred thousand dollars a year…compared to ACTs and RDTs and others”. (Programme/Project Coordinator)*

The WHO policy recommendations, based on a robust evidence review, also facilitated smooth policy transition. Such a consensus makes it easier for countries to adopt as stipulated below:

*“WHO has also been pushing that we change… and the policy brief was backed by a lot of scientific evidence that was done extensively across the globe… so that push from these global stakeholders also enabled us to work fast”. (Programme/Project Coordinator)*

*“Actually we just adapted the WHO guidelines…revised the malaria treatment guidelines accordingly…that was really straight forward”. (Researcher/Advisor)*

The NMCP, who are the key stakeholders in implementing malaria control interventions in the country, had a vested interest in updating the policy. The NMCP made all efforts from within the government to adopt and implement the policy change. This was confirmed below:

*“Overall the National Malaria Control Programme who are the mandated programme to look at malaria, also had keen interest for this to happen and be implemented”. (Programme/Project Coordinator)*

The inclusion of relevant stakeholders at the beginning of the process of change was key in driving the policy update. The inclusion of RHD, who implement IPTp as an integrated reproductive health service, was strategic at policy implementation stage, as described below:

*“I need to point out that the reproductive health directorate [as a] key department in malaria in pregnancy issues were also very supportive…you know we cannot talk of malaria in pregnancy without the reproductive health directorate because [it] is a platform that we use to implement IPTp”. (Programme/Project Coordinator)*

### Challenges encountered in the policy process

The NMCP were mandated to develop the new IPTp-SP as a stand-alone malaria control policy. The previous IPTp guidelines were embedded as a component within the malaria treatment guidelines. Developing new stand-alone IPTp-SP guidelines was a cumbersome process that had to incorporate information that was previously part of the Malaria Treatment guidelines, as describe below:

*“But now malaria in pregnancy were embedded in malaria case management….. and what was lacking in those documents were the detailed health education that goes with it…financing, partnership…all those things were missing because it was only considered as a treatment component not necessarily as a strategy…so the challenge was that we had to develop [the] guidelines from a scratch”. (Programme/Project Coordinator)*

Developing separate guidelines meant that health workers needed to be reoriented to consider IPTp-SP as a preventive strategy and an integrated case management tool. As acknowledged by a stakeholder that it was also difficult to bring together various stakeholders to one gathering and commit their time to developing the guidelines:

*“To get different stakeholders come together and agree on something it takes time because people have got a lot of demands on their work…so for them also to dedicate their time to this, it’s a little bit of time”. (Programme/Project Coordinator)*

Orientation of health workers occurred in stages, one district at a time. Thus other districts were still implementing the old policy while training was taking place. This was partly due to funding partners’ policies to release funds in stages. Concerns were raised as below:

*“This time around we are conducting cascade training not as the way we always do, because things change, partners change the way of doing business, so as we are going down to the districts to do the actual trainings it will be a little bit slow because others are still using the two dose…….because of the way we are implementing due to funders money. But otherwise in a nutshell we just believe that by the end of the year we [will] have finished and the whole country is [will] implement one policy”. (Policy maker)*

In addition to this challenge was the delay in training health workers due to the change in per diem policy by the government on all Developing Partner (DP) programmes in Malawi.

### Lessons learnt during the policy change

Participants highlighted that dedication to the policy process is critical, especially government commitment. Partners can provide resources but if the MOH as owners of health policies are not motivated, the process will face challenges. A stakeholder confirmed this:

*“The most important element for a policy to be effectively developed, the relevant government department should have the interest in that policy and they drive the whole process…. the NMCP team were so keen to have this done…that’s why we didn’t find a lot of problems”. (Programme/Project Coordinator)*

Another important factor highlighted by participants was the availability of local evidence on which to base changes. An advisor revealed that research evidence forms the foundation for further policy changes:

*“Normally when we want to effect a policy change there must have been a study that was conducted or an assessment that was conducted…so that has always been the trend in Malawi that we are guided by studies”. (Researcher/Advisor)*

Participants stressed that resources should be available at all stages from policy adoption to implementation to achieve smooth policy change, as highlighted below:

*“When you are embarking on policy change you should have everything available, you should have the money for the change, for everything that means the drugs themselves, for the guideline change, information to the general public because you can have the money to do the trainings but if you don’t inform the public it is very difficult for them to welcome the intervention quickly. So when you have all this together the policy change is very smooth”. (Policy maker)*

Participants acknowledged the significance of involving relevant stakeholders in the policy process ranging from funding partners, policy makers in the MOH, Policy implementers, and the public.

## Discussion

The reduced efficacy of SP in the treatment of uncomplicated malaria led to its replacement with ACT as recommended by WHO [28]. Inevitably, concerns were raised about the continued use of SP for IPTp. Following these concerns, IPTp-SP was extensively monitored to evaluate its use or explore alternative drugs. Recently a study conducted in Malawi proposing intermittent screening and treatment with dihydroartemisin–piperaquine (ISTp-DP) as an alternative to IPTp-SP did not show superiority in both parasite clearance and birth outcomes. Thus it recommended continued use of SP for IPTp [29]. Whilst alternative drugs or strategies for IPTp have not yet been found, evaluations of IPTp-SP have revealed that giving SP to all pregnant women at each antenatal care visit from early in the second trimester, with subsequent doses spaced four weeks apart up to the time of delivery, is beneficial for birth outcomes [8]. Concerns of increased doses of SP for IPTp were raised on the uptake of folic acid and iron during pregnancy because of SP is a folate antagonist. However, evidence has shown that there is no interference with SP when the right doses of 30–60 mg of element iron plus 0.4 mg/day folic acid supplementation are administered [8, 30].

Comprehensive studies conducted in Malawi have contributed to the body of knowledge on IPTp and have informed local and international IPTp policies. Upon implementation of the first global IPTp-SP policy [6], Malawi immediately started monitoring the policy and confirmed the safety of SP in IPTp and established that multiple doses of SP during pregnancy led to a highly significant reduction in the incidence of LBW [17]. The two SP dose regimen remained unchanged despite these results. Increased *P. falciparum* resistance to SP in the treatment of uncomplicated malaria led to similar concerns regarding the use of SP in IPTp. Several studies monitored the effects of IPTp-SP during pregnancy and birth outcomes. These studies revealed the positive outcomes of IPTp-SP and recommended that more SP doses would have further positive results [14, 18, 19, 21–24]. Despite this evidence originating in Malawi, IPTp-SP policy changes were only made after the amended WHO recommendation was released in 2013. The two studies conducted in Malawi [14, 21] were included in the meta-analysis that led to the change in WHO recommendations [20] which then informed local policy changes. Nevertheless, the WHO does not impose recommendations since countries are at liberty to adopt or adapt them as has been the case for Malawi.

The policy review process in Malawi was largely based on WHO recommendations [8, 15] which incorporated evidence from Malawi for agenda setting [9, 25] and policy development [14, 18, 19, 21–23, 26]. The lack of references in the local policy document [12] hampered the assessment of the degree to which local research was consulted [31]. While the availability of evidence is one content factor that needs to be considered when making a policy [3], the policy process often overlooks actors, processes and contextual factors [4]. In Malawi, the involvement of the right stakeholders during the policy process was strategic. Although the NMCP is the overall coordinating body for malaria interventions, the inclusion of the RHD was vital since they are responsible for delivery of reproductive health services in the country including IPTp-SP. The importance of this collaboration can never be over-emphasised by the malaria in pregnancy working group meeting in Kenya, which attracted both NMCP and reproductive health MOH country representatives to discuss the Roll Back Malaria guidelines for MIP [32]. Funding partners in the process played a major role through tracking progress and obtaining first-hand reports.

Changing the policy was met with technical and administrative challenges that included the tedious process of developing new IPTp-SP guidelines, bringing together all stakeholders in one place, and the cascade training of health workers which was adversely affected by change of per diem policy by the government. The previous per diem policy left room for public funds abuse when among other things events such as training workshop were conducted away requiring participants to claim for some expenses not incurred and it was widely viewed as a method of supplementing one’s salary. The change in policy hence led to boycott of such events until resolutions were sought and one of the logistical issues was resolved by conducting training workshops in areas where most participants resided.

Nonetheless, many opportunities facilitated a smooth policy process. Most important was leadership by the MOH through the NMCP that showed keen interest in seeing the policy developed and implemented. Similarly, the NMCP was identified as critical in driving government efforts in engaging and collaborating with the right stakeholders such as researchers in seeking evidence for policy formulation and its implementation [33]. It is important that the motivation emanates from the government if such a process is to be realized. Policy change in Timor-Leste that contained an escalation of malaria cases during a crisis was similarly supported and driven by government [34]. The use of WHO recommendations as motivation for policy change facilitated support from PMI, who provided technical and funding support to the process.

## Conclusion

Malawi changed its IPTp policy based on the WHO recommendation in 2012. Research conducted in Malawi was instrumental in changing the global IPTp-SP policy due to the inclusion of findings in the systematic review that led to the WHO policy change. Malawi adapted and changed its IPTp-SP policy based on the resulting WHO recommendations. This change did not face many hurdles but was a welcome opportunity to address some of the challenges faced by health workers during implementation of the previous policy. The policy updating process has highlighted the importance of commitment by the concerned government department to be motivated and drive the process. This should be accompanied by a thorough stakeholder analysis to identify and involve relevant key stakeholders from the initial stages of the policy change process. In addition, it is critical to utilize local evidence for this process and address current local health burdens leading to efficient public health care. The local evidence used in the process should be documented in the policy documents and guidelines for purposes of tracking research utilization and its impact.

Ideally, policymakers should use a framework that facilitates the use of malaria research to champion knowledge translation and work towards addressing the malaria burden in Malawi. Therefore, lessons from this study will inform the development of the malaria research-to-policy framework in Malawi and the process of developing this framework can also advise the development of research-to-policy frameworks in other settings.

## Abbreviations

ACT: Artemisin-based Combination Therapy
CHAI: Clinton Health Access Initiative
CQ: Chloroquine
ERG: Evidence Review Group
WHO: World Health Organization
IPTp-SP: Intermittent Preventive Treatment in pregnancy with sulfadoxine–pyrimethamine
KIIs: Key Informant Interviews
LBW: Low Birth Weight
MIP: Malaria in Pregnancy
MOH: Ministry of Health
MPAC: Malaria Policy Advisory Committee
NMCP: National Malaria Control Programme
PMI: Presidential Malaria Initiative
RHD: Reproductive Health Directorate
SSDI-services: Support for Service Delivery Integration-Services
TOTs: Trainer of Trainers
USAID: United States Agency for International Development
